# Non-coding RNAs Shaping Muscle

**DOI:** 10.3389/fcell.2019.00394

**Published:** 2020-02-07

**Authors:** Julie Martone, Davide Mariani, Fabio Desideri, Monica Ballarino

**Affiliations:** ^1^Department of Biology and Biotechnology Charles Darwin, Sapienza University of Rome, Rome, Italy; ^2^Center for Human Technologies, Italian Institute of Technology, Genoa, Italy

**Keywords:** myogenesis, non-coding RNAs, long non-coding RNAs, lncRNAs, circular RNAs, circRNAs, Piwi-interacting RNAs, transfer RNA-derived fragments

## Abstract

In 1957, Francis Crick speculated that RNA, beyond its protein-coding capacity, could have its own function. Decade after decade, this theory was dramatically boosted by the discovery of new classes of non-coding RNAs (ncRNAs), including long ncRNAs (lncRNAs) and circular RNAs (circRNAs), which play a fundamental role in the fine spatio-temporal control of multiple layers of gene expression. Recently, many of these molecules have been identified in a plethora of different tissues, and they have emerged to be more cell-type specific than protein-coding genes. These findings shed light on how ncRNAs are involved in the precise tuning of gene regulatory mechanisms governing tissues homeostasis. In this review, we discuss the recent findings on the mechanisms used by lncRNAs and circRNAs to sustain skeletal and cardiac muscle formation, paying particular attention to the technological developments that, over the last few years, have aided their genome-wide identification and study. Together with lncRNAs and circRNAs, the emerging contribution of Piwi-interacting RNAs and transfer RNA-derived fragments to myogenesis will be also discussed, with a glimpse on the impact of their dysregulation in muscle disorders, such as myopathies, muscle atrophy, and rhabdomyosarcoma degeneration.

## Introduction

In the last 50 years, RNA has been subjected to an unprecedented revaluation that gradually shifted the perspective on how gene expression is regulated from a coding to a non-coding point of view. With the discovery of messenger RNA (mRNA) codon–transfer RNA (tRNA) anticodon interaction, tRNAs have become forerunners of non-coding functionality (Hoagland et al., 1958; Steitz and Jakes, 1975). In roughly 30 years of experiments and observations, novel classes of non-coding RNAs (ncRNAs), such as ribosomal RNAs (rRNAs) (Scherrer and Darnell, 1962), small nuclear RNAs (Weinberg and Penman, 1968), small nucleolar RNAs (Reddy and Busch, 1988), and microRNAs (miRNAs) (Lee et al., 1993; Lagos-Quintana et al., 2001; Lau et al., 2001), attracted the attention of the scientific community by showing their participation to several biological processes. A significant contribution to these discoveries was made by the advances in both sequencing technologies and data open sharing, which brought the new millennium into the “omics” revolution. In this context, the identification and the extraordinary cell-type-specific expression of new classes of ncRNAs, including long ncRNAs (lncRNAs) and circular RNAs (circRNAs), provided new clues into the understanding of tissue homeostasis (Kapranov et al., 2007; Mattick, 2011; Nagano and Fraser, 2011; Hon et al., 2017).

LncRNAs are arbitrarily defined as transcripts longer than 200 nucleotides, which regulate gene expression at transcriptional and post-transcriptional level (Rinn and Chang, 2012; Ulitsky and Bartel, 2013; Fatica and Bozzoni, 2014). The function of these transcripts is in close connection with their specific subcellular localization, ranging from an almost exclusive nuclear (Heard and Disteche, 2006; Tripathi et al., 2010; Chen et al., 2016) or cytoplasmic (Cesana et al., 2011; van Heesch et al., 2014) enrichment, up to a more uniform and less defined distribution (Ballarino et al., 2015; Alessio et al., 2019). Together with lncRNAs, it is nowadays well-established that cells also express circRNAs. These are endogenously expressed and covalently closed single-stranded RNA species that derive from back-splicing circularization events (Jeck et al., 2013; Chen, 2016). Differently from linear lncRNA molecules, the lack of 5′- and 3′-ends confers to circRNAs greater stability and amplifies their chance to exert various biological tasks. As a common aspect, the function of both lncRNA and circRNA is determined by their ability to recognize nucleic acids by base pairing with their high versatility to interact with proteins (Guttman and Rinn, 2012; Rinn and Chang, 2012; Batista and Chang, 2013). For this reason, the post-genomic epoch has been marked by the establishment of innovative technologies, which have been extremely helpful for identifying the ncRNA interactome, thus providing crucial clues on their mechanisms of action (Ule et al., 2003; Licatalosi et al., 2008; Engreitz et al., 2014; McHugh and Guttman, 2018). The RNA antisense purification (RAP) is one of the most RNA-centric leading-edge approach able to purify and identify the interacting partners of a specific RNA (McHugh and Guttman, 2018). The protein-centric mirror technique is represented by the cross-linking and immunoprecipitation (CLIP) assay (Ule et al., 2003; Licatalosi et al., 2008). Both the systems exploit the ultraviolet (UV) cross-linking to create covalent linkages between directly interacting RNAs and proteins, purifying the molecule of interest under stringent conditions to reduce background signals. In addition, a method to systematically map RNA–RNA interactions, based on psoralen cross-linking, has been also developed (Engreitz et al., 2014).

To date, the vast majority of studies have focused on the role of miRNAs in muscle development (Ballarino et al., 2016; Wang J. et al., 2018; Colpaert and Calore, 2019). In this article, we highlight the importance of some less studied classes of ncRNAs, such as lncRNAs and circRNAs, focusing on their function in skeletal and cardiac muscles biology. We discuss paradigmatic examples that support their nuclear and cytoplasmic activities and report the latest findings of lncRNAs and circRNAs containing functional open reading frames (ORFs) (Chekulaeva and Rajewsky, 2019). In the final section, we also provide a broad overview on other classes of ncRNAs [i.e., Piwi-interacting RNAs (piRNAs) and tRNA fragments], which are well-known for their canonical functions but only recently emerged as functional in muscle physiology (Liapi et al., 2019).

## lncRNAs in Skeletal and Cardiac Myogenesis

Since the establishment of the murine myoblast C_2_C_12_ cell line, which allowed to reproduce *in vitro* the different stages of myogenic differentiation (Yaffe and Saxel, 1977), and the discovery of MYOD1 as the “master gene” for myogenesis (Davis et al., 1987), a wealth of knowledge has been accumulated regarding the ncRNA-mediated regulatory networks governing muscle biology. Indeed, multiple examples of nuclear (Table 1) and cytoplasmic (Table 2) non-coding transcripts involved in the acquisition of both skeletal and cardiac muscle identity have been detailed over the last decade, with an increasing degree of emphasis on large species (Neguembor et al., 2014; Rotini et al., 2018).

**Table 1:** Nuclear long non-coding RNAs (lncRNAs) in skeletal and cardiac myogenesis.

**lncRNA**	**Species**	**Function/mechanism**	***In vivo* model phenotype**	**Expression**	**References**
Upperhand (UPH)	Mouse, human	Its transcription is required to establish a permissive chromatin environment at Hand2 enhancer locus	Embryonic lethality and heart failure	Highly expressed in heart	Anderson et al., 2016
Handsdown (HDN)	Mouse, human	Its transcription is required to regulate *in cis* the production of Hand2	Embryonic lethality and defects in uterine implantation	Expressed in early developing heart	Ritter et al., 2019
Braveheart (BVHT)	Mouse	It is required for the activation of a core cardiovascular gene network by preventing SUZ12 to repress MesP1 promoter	Not available	Highly expressed in heart	Klattenhoff et al., 2013
Fendrr	Mouse, human	Binds PRC2 and TrxG/MLL to influence histone marks on lateral mesoderm specific gene promoters	Embryonic lethality	Lateral mesoderm-specific expression	Grote et al., 2013
SYISL	Mouse	Promotes cellular proliferation by inhibiting muscle-specific transcription factors through an EZH2-recruitment mechanism	Defects in muscle fiber density and muscle mass	Highly expressed in muscle	Jin et al., 2018
Neat1	Mouse, human	Sustains myoblast proliferation and blocks differentiation by recruiting EZH2 to muscle-specific promoters	Defects in muscle regeneration	Expressed in a wide range of tissues	Wang S. et al., 2019
SRA	Mouse, human	It is required for proper cell differentiation by coactivating MyoD together with the RNA elicase p68/p72	Not available	Expressed in a wide range of tissues	Caretti et al., 2006; Hubé et al., 2011
CE	Mouse	Acts as enhancer RNA to increase RNA POL II occupancy at MyoD locus	Not available	Expressed in myogenic lineage	Mousavi et al., 2013
DRR	Mouse	Acts as enhancer RNA to activate MyoD downstream myogenic genes	Not available	Expressed in myogenic lineage	Mousavi et al., 2013
RAM	Mouse, human	Promotes the activation of the myogenic program by binding to MyoD and supporting the assembly of MyoD-Baf60c-Brg complex	Defects in muscle regeneration	Skeletal muscle-specific	Yu et al., 2017
Dum	Mouse, human	Promotes myoblasts differentiation by recruiting Dnmts to regulate Dppa2 expression	Defects in muscle regeneration	Highly expressed in muscle	Wang et al., 2015
YY1	Mouse, human	Activates gene expression *in trans* by interacting with YY1 and removing YY1/PRC2 complex from target promoters	Downregulation of keys myogenic genes	Highly expressed in muscle	Zhou et al., 2015
IRM	Mouse	Regulates the expression of myogenic genes by binding to MEF2D and promoting the assembly of MyoD/MEF2D	Impairment of muscle regeneration	Expressed in brain and skeletal muscle	Sui et al., 2019
Myolinc	Mouse	Promotes *in vitro* myogenesis by recruiting TDP-43 on muscle-specific targets both *in cis* and *in trans*	Defects of regeneration in muscle cells	Highly expressed in heart and skeletal muscle	Militello et al., 2018
Myoparr	Mouse, human	Induces myoblasts cell cycle withdrawal and activates myogenin transcription by interacting with MyoD coactivator Ddx17	Prevention of atrophy in denervated muscle	Skeletal muscle-specific	Hitachi et al., 2019a,b
PVT1	Mouse	Interacts to and stabilizes c-Myc impacting the activity of Bcl-2	Defects in mitochondrial respiration and morphology, apoptosis, and myofiber size	Highly expressed in skeletal muscle	Alessio et al., 2019
Myheart (Mhrt)	Mouse, human	Antagonizes the function of the transcription factor Brg1 preventing the recognition of its genomic targets	Mhrt restoration improves cardiac function in TAC-stressed hearts	Highly expressed in heart	Han et al., 2014
Chronos	Mouse	Represses Bmp7 by recruiting EZH2 on its promoter	Myofibers hypertrophy	Highly expressed in heart and skeletal muscle	Neppl et al., 2017
MEG3	Mouse, human	Controls cardiac fibrosis through the regulation of matrix metalloproteinase-2 production	Decreases cardiac fibrosis and improves diastolic performance	Highly expressed in cardiac fibroblasts during pressure-overload heart remodeling	Piccoli et al., 2017; Wu et al., 2018
Charme	Mouse, human	Acts as a chromatin architect to promote myoblasts differentiation	Cardiac remodeling phenotype at developmental onset	Skeletal muscle and heart-specific	Ballarino et al., 2018

**Table 2:** Cytoplasmatic long non-coding RNAs (lncRNAs) in skeletal and cardiac myogenesis.

**lncRNA**	**Species**	**Function/mechanism**	***In vivo* model phenotype**	**Expression**	**References**
LNC-31	Mouse, human	Promotes ROCK1 translation by stabilizing YB-1 protein	Not available	Expressed in a wide range of tissues	Ballarino et al., 2015; Dimartino et al., 2018
LiNC-MD1	Mouse, human	ceRNA for miR-133 and miR-135 to regulate the expression of MAML1 and MEF2C	Not available	Muscle-specific expression	Cesana et al., 2011
LNC-MG	Mouse	ceRNA for miR-125b and miR-351-5p to control insulin-like growth factor 2 protein abundance and regulate lactamase β expression	Muscle atrophy and loss of muscular endurance	Skeletal muscle enriched	Zhu et al., 2017; Du et al., 2019
AK017368	Mouse	ceRNA for miR-30c to regulate trinucleotide repeat containing-6A	Muscle hypertrophy	Enriched in lung, heart, and skeletal muscle	Liang et al., 2018
LNC-MUMA	Mouse, human	ceRNA for miR-762 to regulate MyoD abundance	Its overexpression reverses muscle atrophy	Skeletal muscle enriched	Zhang et al., 2018a
MAR1	Mouse	ceRNA for miR-487b to regulate Wnt5a protein	Its overexpression increases muscle mass and strength	Skeletal muscle-enriched	Zhang et al., 2018b
LNC-MYOD	Mouse, human	Regulates the translation of N-Ras and c-Myc by sequestering IMP2 protein	Not available	Myoblasts and early myotubes specific	Gong et al., 2015
ATROLNC-1	Mouse	Interacts to and inhibits ABIN-1 protein to increase MuRF-1 expression	Attenuates muscle wasting	Highly expressed in skeletal muscle, upregulated in atrophying muscles	Sun et al., 2018
ZFAS1	Mouse, human	Binds to and inhibits SERCA2a protein affecting the Ca^2+^ transient dynamics	Restores heart contraction parameters in MI animals	Highly expressed in different cancers and MI	Zhang et al., 2018c; Jiao et al., 2019
DACH1	Mouse, human	Binds to and inhibits SERCA2a protein affecting the Ca^2+^ transient dynamics	Heart failure	Upregulated upon heart failure	Cai B. et al., 2019
CTBP1-AS2	Mouse, rat	Binds FUS to induce a non-physiological stabilization of TLR4 mRNA	Attenuates cardiomyocytes hypertrophy	Upregulated in cancer and cardiomyocyte hypertrophy	Luo et al., 2019

### Nuclear-Enriched lncRNAs

Aside from the “finished” non-coding transcript, the *act* of transcription has been proposed to be functional in myogenesis. Few years ago, Anderson et al. (2016) reported that the transcription of Hand2-associated lncRNA, named Upperhand (Uph) is required to establish a permissive chromatin environment at the Hand2 enhancer locus. Indeed, blockade of Uph transcription, but not the knockdown of the mature transcript, abolished Hand2 expression and caused heart failure and embryonic lethality in mice. In a very recent paper, Ritter et al. identified a novel lncRNA locus named Handsdown (Hdn) that is fundamental for the transcriptional regulation of Hand2 gene (Ritter et al., 2019). *In situ* hybridization of both Hdn and Hand2 demonstrates that the transcripts are coexpressed in the same cells or at least in the same parts of the tissue at different time points of embryonic heart specification. Interestingly, Hdn knockout shows that the locus is essential for embryonic development and uterine implantation, while its reduced expression in the heterozygous mice is haploinsufficient for proper heart formation. Chromosome conformation capture (Hi-C and 4C) analyses demonstrate that Hdn expression is crucial for structuring the genomic region and regulating *in cis* Hand2 production in a transcript-independent, transcription-based manner.

Upon their transcription, most of the nuclear-enriched lncRNAs regulate myogenesis by recruiting chromatin-modifying complexes to nearby (*in cis*) or distant (*in trans*) genomic loci. The Polycomb repressive complex 2 (PRC2), which catalyzes the methylation of lysine 27 on histone H3, is one of the most studied chromatin-modifying complexes found to be recruited by myogenic lncRNAs. Earlier studies from 2013 described two paradigmatic examples, which include Braveheart (Klattenhoff et al., 2013) and Fendrr (Grote et al., 2013). Overall, these findings highlighted the functional importance of lncRNA-based epigenetic mechanisms in the regulation of cell fate and greatly contributed to advance our understanding of the regulatory networks driving cardiac lineage commitment. In skeletal muscle, a more recent study (Jin et al., 2018) reported the identification of SYISL (SYNPO2-intron sense-overlapping), an abundant and intron-encoded lncRNA whose expression increases with C_2_C_12_ myoblasts differentiation. Mechanistically, SYISL promotes proliferation (and inhibits differentiation) by silencing the muscle-specific expression of myogenin, muscle creatine kinase, and myosin heavy chain (Myh4) through the recruitment of the PRC2 subunit enhancer of Zeste homolog 2 (EZH2), to their promoters. Similarly, Wang S. et al. (2019) described a functional interaction between EZH2 and Neat1 in myogenic cells. Nuclear Enriched Abundant Transcript 1 (Neat1) is a nuclear lncRNA essential for paraspeckles formation, stability, and integrity (Souquere et al., 2010), which exerts critical roles in several biological processes as well as in tumorigenesis and non-cancerous diseases (Ghafouri-Fard and Taheri, 2019; Prinz et al., 2019). During C_2_C_12_ differentiation, Neat1 sustains proliferation and blocks differentiation by recruiting EZH2 to p21 (cyclin-dependent kinase inhibitor 1 A) and to muscle specific promoters (i.e., Myog, Myh4, and Tnni2). To note, Neat1 depletion *in vivo* was shown to delay muscle regeneration induced by cardiotoxin treatment.

Besides the recruitment of PRC2, several nuclear-enriched lncRNAs regulate the binding of transcription factors and transcriptional coactivators to specific myogenic loci. Several of these lncRNAs, such as SRA (Caretti et al., 2006; Hubé et al., 2011), the CE and DRR endogenous RNAs (eRNAs) (Mousavi et al., 2013), and Linc-RAM (Yu et al., 2017) regulate the activity of the master transcription factor MyoD. Others impinge myogenesis either through the binding of specific myogenic factors or transcription regulators (Dum, Wang et al., 2015; Linc YY1, Zhou et al., 2015). Irm, a lncRNA that regulates myogenic genes expression in C_2_C_12_ cells by directly binding to MEF2D (Sui et al., 2019), belongs to the first category. A recent example from the second group includes Myolinc (AK142388), a lncRNA that promotes myoblasts fusion by the recruitment of the TAR DNA-binding protein 43 (TDP-43) *in cis*, on the neighboring Filip1 promoter, and *in trans*, on muscle-specific targets (Militello et al., 2018). As for Neat1, Myolinc knockdown causes a delayed regeneration of skeletal muscle in adult mice. Hitachi et al. recently described Myoparr, a promoter-associated lncRNA derived from the upstream region of the myogenin gene, which has a dual role in myogenesis: on the one hand, it is involved in myoblasts cell cycle withdrawal by the transactivation of myo-miRNAs expression; on the other hand, it is responsible for the activation *in cis* of myogenin transcription, which allows the entrance of myoblasts into myogenic differentiation. The latter is mediated by the interaction between Myoparr and the MyoD-coactivator Ddx17, which results in the RNA-Pol II recruitment on myogenin promoter and in transcriptional activation (Hitachi et al., 2019a). In a subsequent paper, the same authors also examined the role of Myoparr in skeletal muscle atrophy. They found that, in denervated muscles, Myoparr knockdown prevents atrophy by activating the bone morphogenetic protein signaling and by increasing the expression of GDF5, a muscle atrophy inhibitor. The existence of a Myoparr human counterpart makes the lncRNA potentially eligible as therapeutic target for neurogenic atrophy (Hitachi et al., 2019b).

Like Myoparr, it is not uncommon to find association between dysregulation of lncRNA expression and pathological conditions that induce changes in muscle mass, such as atrophy, hypertrophy, and cardiac remodeling. Another example is Pvt1, an evolutionary conserved lncRNA whose upregulation in cancer cells influences positively the stability of the oncoprotein transcription factor MYC (Tseng and Bagchi, 2015). A similar Pvt1/MYC interplay also plays a role during muscle atrophy, in which the Pvt1-mediated c-Myc stabilization impacts on the activity of Bcl-2, a crucial regulator of apoptosis and autophagy (Alessio et al., 2019). First evidence on the involvement of lncRNAs in hypertrophy came in 2014 from Chang's lab, which described a reduced hypertrophic growth and improved cardiac functions upon restoration of myosin heavy-chain-associated RNA transcripts (Mhrt) levels in transverse aortic constriction (TAC)-stressed hearts (Han et al., 2014). Analogously to Mhrt, in skeletal muscle cells, the Gm17281 lncRNA, known as Chronos, inhibits hypertrophy through the Bmp7 signaling. Mechanistically, Chronos acts as an epigenetic repressor of Bmp7 cascade through the recruitment of EZH2 on the Bmp7 promoter (Neppl et al., 2017). In murine cardiac fibroblasts, a microarray analysis led to the identification of several lncRNAs dysregulated after pressure overload upon TAC. Among the most abundant nuclear species, Piccoli et al. found the lncRNA maternally expressed gene 3 (Meg3). In cardiac fibroblasts, Meg3 controls the production of the matrix metalloproteinase-2 and, consequently, cardiac fibrosis (Piccoli et al., 2017). Indeed, Meg3 silencing was shown to reduce diastolic dysfunction and fibrosis by preventing the pathological induction of the matrix metalloproteinase-2 protein after pressure overload. Prospectively, the inhibition of Meg3 expression might represent a conceivable strategy to prevent the development of fibrosis and cardiomyocytes hypertrophic growth observed in heart diseases. The finding that Meg3 expression is upregulated in clinical heart failure samples and controls the apoptosis of human cardiomyocites (Wu et al., 2018) makes Meg3 particularly attracting as a therapeutic target. *Charme* (chromatin architect of muscle expression) is another nuclear-enriched lncRNA (Ballarino et al., 2018), whose ablation *in vivo* leads to a pronounced phenotype of cardiac remodeling at developmental onset. In myotubes, *Charme* contributes to the chromatin proximity between important myogenic loci, thus controlling their expression at transcriptional level. This epigenetic control is in line with emerging studies indicating that lncRNAs can act as modular scaffolds to shape the formation of chromosome territories where coregulated gene expression occurs (Clemson et al., 2009; Tripathi et al., 2010; Engreitz et al., 2013; Hacisuleyman et al., 2014; Ribeiro et al., 2018). Moreover, the existence of a *Charme* ortholog transcript produced by the human syntenic locus makes this lncRNA an appealing target for cardiovascular diseases.

### Cytoplasmic-Enriched lncRNAs

In the cytoplasm (Table 2), lncRNAs mainly act as regulators of mRNA stability and translation. These activities depend on two major lncRNA peculiarities: (i) the capacity to base pair with RNA counterparts and (ii) the ability to act as flexible scaffolds for tethering RNAs and proteins and ensure their concerted action. Paradigmatic examples include the antisense lncRNAs known as half-STAU1-binding site RNAs (1/2-sbsRNAs) that induce Staufen1-mediated (STAU-mediated) mRNA decay (Wang J. et al., 2013). A new identified mechanism by which lncRNA–mRNA–protein interplay exerts a role in skeletal myogenesis is represented by lnc-31, a lncRNA controlling the maintenance of myoblast proliferation both in murine and human myoblasts (Ballarino et al., 2015). A key determinant for lnc-31 function is the presence of a 22nt-long region, which binds the 5′ untranslated region of ROCK1 mRNA and positively controls protein synthesis. The concomitant recruitment and the consequent stabilization of the translational activator Y-box protein 1 (YBX1) by lnc-31 concur to this translational control, as demonstrated by the decrease in ROCK1 protein upon Y-box protein 1 (YBX1) knockdown in proliferating myoblasts (Dimartino et al., 2018).

Another mechanism which underlies the capacity of lncRNAs to influence mRNA stability is based on their ability to act as competing endogenous RNAs (ceRNAs) for miRNAs (Cesana et al., 2011; Kallen et al., 2013; Han et al., 2015; Yan et al., 2015) or proteins (Gong et al., 2015). Since the discovery of linc-MD1, which was one of the first miRNA sponges to be identified, additional muscle relevant ceRNAs have been described. Recent examples include lnc-mg (Zhu et al., 2017), AK017386 (Liang et al., 2018), LncMUMA (Zhang et al., 2018a), and MAR1 (Zhang et al., 2018b) lncRNAs. Lnc-mg is a 5′-capped and polyadenylated ncRNA whose ablation (i) *in vitro*, reduces the capacity of murine satellite cells (MuSCs) to differentiate and (ii) *in vivo*, results in muscular atrophy associated to reduced muscle endurance. Mechanistically, lnc-mg acts as a miRNA-125b sponge to increase the abundance of insulin-like growth factor 2, an already known miRNA-125b target (Ge et al., 2011). More recently, lnc-mg was shown to act as a molecular sponge for miR-351-5p, which functions in skeletal myogenesis by targeting lactamase β (Du et al., 2019). AK017368 is a muscle highly enriched lincRNA able, to induce proliferation and inhibit myoblasts differentiation *in vitro*. AK017368 depletion in murine muscles induce fibers hypertrophy. Liang et al. (2018) demonstrated that AK017368 acts as a sponge for miR-30c by competing with its known mRNA target Tnrc6a (trinucleotide repeat containing-6A), already involved in control of myogenic differentiation by guiding Ago protein into the nucleus to lead miRNA-mediated gene silencing (Nishi et al., 2013). Another example of ceRNA promoting myogenesis is LncMUMA, a mechanical unloading-induced muscle atrophy-related lncRNA functioning as a sponge for miR-762 to regulate *in vitro* MyoD abundances (Zhang et al., 2018a). As LncMUMA enforced expression reverses the established muscle atrophy in hindlimb suspension mice, the above studies provide a novel therapeutic targeting for treating muscle atrophy following mechanical unloading. The same authors identified muscle anabolic regulator 1 (MAR1), a lncRNA that acts as miR-487b sponge to regulate Wnt5a protein, an important regulator of myogenesis (Tajbakhsh et al., 1998) and a known target of mir-487b in other cellular systems (Xi et al., 2013). MAR1 resulted to be positively correlated with muscle differentiation and growth, both *in vitro* and *in vivo*. Moreover, muscle mass and strength were increased in MAR1 overexpressing condition, suggesting a putative therapeutic role for muscle atrophy treatment.

Among the lncRNAs that are able to modulate the function of cytoplasmic proteins, good examples are lncMyoD (Gong et al., 2015) and Atrolnc-1 (Sun et al., 2018). LncMyoD is directly activated by MyoD at the onset of differentiation and negatively regulates the translation of N-Ras and c-Myc by directly binding and sequestering the IMP2 (insulin-like growth factor 2–mRNA-binding protein 2) factor. Atrolnc-1 was recently identified to be highly expressed in atrophying muscle from mice with cachexia. The authors demonstrated that Atrolnc-1 predominantly interacts with cytoplasmic proteins, and, in particular, they focused on A20 binding inhibitor of nuclear factor kappa B-1 (NF-κB-1), an inhibitor of IκB degradation and NF-κB activation (Mauro et al., 2006; Hooper et al., 2014). The consequence of this cytoplasmic interaction is the inhibition of A20 binding inhibitor of NF-κB-1 function, leading to an increased activity of NF-κB that in turn increases the expression of the ubiquitin E3-ligase MuRF-1 (Rom and Reznick, 2016). Moreover, the overexpression of Atrolnc-1 in wild-type muscles causes increased MuRF-1 expression, which results in myofiber atrophy.

In the heart, cytoplasmic lncRNAs have also been proposed as regulators of the calcium reuptake occurring after cardiac muscle contraction. ZFAS1 lncRNA is markedly upregulated in both mouse and human cardiac tissues subjected to myocardial infarction (MI). While its knockdown restores heart contraction parameters to normal levels in a MI mouse model, its overexpression in wild-type mice induces a MI-like phenotype. Mechanistically, ZFAS1 directly binds the SERCA2a protein, which is responsible for calcium reuptake in the sarcoplasmic reticulum, and it is responsible for the downregulation of the protein levels, thus affecting the Ca^2+^ transient dynamics (Zhang et al., 2018c). More recently, the same group described a similar mechanism for DACH1 lncRNA. This RNA molecule is upregulated in failing hearts of mice and humans and directly interacts with SERCA2a; its overexpression is associated with an augmented ubiquitination of the protein, driving it to proteasome degradation (Cai B. et al., 2019).

As described for nuclear lncRNAs, also the dysregulation of cytoplasmic lncRNAs expression can result in cardiac hypertrophy. For instance, the murine CTBP1-AS2 lncRNA is selectively upregulated in hypertrophic hearts and in Ang-II treated neonatal rat ventricular myocytes. In the cytoplasm, CTBP1-AS2 interacts with the RNA-binding protein fused in sarcoma (FUS) forming a ribonucleoparticle that includes also TLR4 mRNA. Both CTBP1-AS2 and FUS participate in the anomalous stabilization of TLR4 mRNA, which encodes for a well-known driver of the inflammation process at the base of cardiac hypertrophy (Luo et al., 2019).

## lncRNA-Derived Micropeptides

Recently, the functional role of lncRNAs has expanded due to their ability to generate bioactive micropeptides (Table 3), which have been identified mainly in muscle-related functions and cancer development (Ji et al., 2015). The identification of small ORFs that are translatable and encode short peptides remains a major challenge. Two complementary approaches are normally used to discover functional small ORFs: (i) the computational one, based on bioinformatic predictions and (ii) the experimental one, that uses transcriptional and translational data. Several computational tools have been developed to estimate the coding potential of novel identified transcripts. Among the most utilized CPC (Kong et al., 2007) and its updated version CPC2 (Kang et al., 2017), CPAT (Wang L. et al., 2013), COME (Hu et al., 2017), LncRNApred (Pian et al., 2016), PORTRAIT (Arrial et al., 2009), CONC, and others can be cited. The experimental approach is mainly based on the genome-wide translatome that has been studied by ribosome footprinting, a technique introduced by Ingolia in 2009 (Ingolia et al., 2009). The analyses of ribosome dynamics during translation allows the identification of actively translated ORFs (Bazzini et al., 2014) including the ones deriving from previously non-protein coding annotated transcripts. Recently, by applying this technique to human hearts, 169 lncRNAs and 40 circRNAs encoding for previously unknown microproteins have been identified and *in vivo* validated (van Heesch et al., 2019); among them, already known microproteins such as DWORF (Nelson et al., 2016) and SPAR (Matsumoto et al., 2017) were detected (see below). Interestingly, the identified microproteins were associated to cellular processes mainly related to mitochondria. The identification of micropeptides led to hypothesize a dual function for those lncRNAs that were previously characterized for their non-coding functions. In the van Heesch et al. paper, the authors identified 27 human and 5 murine small ORFs in lncRNAs whose non-coding function was already assessed. Among the 169 identified micropeptides deriving from lncRNAs, 16 were cardiac or skeletal muscle specific, suggesting that the list of lncRNA-derived micropeptides, important for muscle physiology, will grow in the coming years. Among the ones that have been already characterized, myoregulin (MLN) was the first transmembrane microprotein identified; it takes origin from a previously annotated muscle-specific lncRNA (NR_038041). The small ORF, located in the third exon of the lncRNA, encodes for a 46-amino-acid long micropeptide. Olson's group showed that this single transmembrane alpha helix peptide interacts with the sarcoplasmic reticulum Ca^2+^-ATPase (SERCA) and impedes Ca^2+^ uptake into the sarcoplasmic reticulum, thus influencing muscle relaxation. According to this observation, MLN showed a strong structural resemblance with the sarcolipin (SLN) and phospholamban (PLN) micropeptides, already known to regulate Ca^2+^ pump activity by directly interacting with SERCA. MLN is the most abundant micropeptide in adult skeletal muscles. For this reason, the authors suggested MLN as a dominant regulator of SERCA activity. Overexpression of MLN peptide caused a reduction in Ca^2+^ uptake while MLN-null mice significantly enhanced Ca^2+^ handling and improved exercise performance (Anderson et al., 2015). A couple of years later, Zhou's lab described a different nuclear non-coding function for the same transcript (NR_038041) that was named Linc-RAM (see above) (Yu et al., 2017). More recently, the same lab extended this work by showing that the fibroblast growth factor FGF2 mediates self-renewal and differentiation of satellite cells by inhibiting Linc-RAM expression in a MyoD-dependent manner (Zhao et al., 2018). Linc-RAM represents a clear case of a non-coding RNA with a well-characterized dual role: one in the nucleus, as non-coding transcript, and the other in the cytoplasm as protein. DWORF (Dwarf Open Reading Frame) is another example of microprotein transcribed from a previously annotated muscle-specific lncRNA. It is highly expressed in the heart and is the third smallest full-length protein annotated in the mouse genome (34 amino acids long). In their paper, Nelson et al. (2016) showed how this peptide, conserved in vertebrates, is able to increase in mouse SERCA pump activity by displacing the already cited inhibitory peptides phospholamban, sarcolipin, and MLN. Hearts from DWORF-overexpressing mice exhibited an increased activity of SERCA, indicating a higher affinity for Ca^2+^, while slow skeletal muscles from DWORF-KO mice showed a reduced SERCA activity with a delayed Ca^2+^ clearance and muscle relaxation.

**Table 3:** Functional long non-coding RNA (lncRNA)-derived micropeptides in skeletal and cardiac myogenesis.

**Micropeptide**	**Species**	**Function/mechanism**	***In vivo* model phenotype**	**Expression**	**References**
Myoregulin (MLN)	Mouse, human	Interacts with SERCA protein and impedes Ca^2+^ uptake into the sarcoplasmic reticulum	Enhances Ca^2+^ handling and improves exercise performance	Skeletal muscle-specific expression	Anderson et al., 2015
DWORF	Vertebrates	Increases SERCA activity by displacing other inhibitory micropeptides	Delays Ca^+^ clearance and muscle relaxation	Muscle-specific expression	Nelson et al., 2016
Mitoregulin (MRLN)/MOXI/ MPM	Mouse, human	Regulates mitochondrial physiology and impairs fatty acid β-oxidation by interacting with different complexes	Smaller skeletal muscle fibers, reduced capacity for exercise, compromised muscle regeneration	Muscle enriched	Makarewich et al., 2018; Stein et al., 2018; Chugunova et al., 2019; Lin et al., 2019
SPAR	Mouse, human	Interacts with the v-ATPase proton pump complex to negatively regulate mTORC1	Promotes skeletal muscle regeneration	Highly expressed in lung, heart and skeletal muscle	Matsumoto et al., 2017
Myomixer/Minion/ Myomerger	Vertebrates, invertebrates	Essential for muscle formation during embryogenesis, satellite cells fusion, and muscle regeneration	Perinatal lethality	Muscle-specific expression	Bi et al., 2017, 2018; Quinn et al., 2017; Zhang et al., 2017; Leikina et al., 2018

In mouse, 1500011K16Rik (LINC00116 in human) is a recently identified lncRNA enriched in heart and skeletal muscle tissues and highly coexpressed with mitochondrial genes. Two papers, published at the same time by different groups (Makarewich et al., 2018; Stein et al., 2018), demonstrated that this lncRNA contains, within its first exon, a nucleotide region that encodes for a predicted conserved single-pass transmembrane microprotein (56 amino acids long) named as mitoregulin (Mtln) because of its inner mitochondrial membranes' localization (Stein et al., 2018) or micropeptide regulator of β-oxidation (MOXI) because of its role in fatty acid β-oxidation (Makarewich et al., 2018). Stein et al. revealed its involvement in supporting mitochondrial high-molecular-weight supercomplexes assembly and/or stability. They demonstrated the high affinity between Mtln and cardiolipin, a phospholipid important for the maintenance of the integrity on the inner mitochondrial membrane. Makarewich et al. showed the ability of the MOXI micropeptide to interact with the mitochondrial trifunctional protein (MTP), an enzymatic complex that catalyzes the last three steps of long-chain fatty acids oxidation, and both groups suggested that Mtnl/MOXI-KO mice present an impaired fatty acid β-oxidation upon increased metabolic demand, probably due to inefficient mitochondrial complex (ri) organization after metabolic switches. Overexpression in HeLa cells and Mtln-KO mice observation allowed to unravel its ability to influence other mitochondrial processes such as membrane potential, Ca^2+^ retention capacity, and oxidative stress (Stein et al., 2018). Moreover, Makarewich et al. observed mitochondrial abnormalities in the KO mice. The role of this micropeptide in mitochondrial respiration was confirmed in a work that was under review when the previous two were published (Chugunova et al., 2019). In this paper, the authors demonstrated that the interaction of Mtln with NADH-dependent cytochrome b5 reductase stimulates the activity of mitochondrial respiratory chain complex I by providing a favorable lipid composition of the membrane. The role of Mtln in promoting myogenic differentiation, muscle growth, and regeneration was further studied by Lin et al., which refers to LINC00116 with the name of micropeptide in mitochondria (MPM, Lin et al., 2019).

The small regulatory polypeptide of amino acid response (SPAR) is another polypeptide translated from a lncRNA (LINC00961) that is conserved among species and highly expressed in heart, skeletal muscle, and lung (Matsumoto et al., 2017). SPAR is characterized by a conserved transmembrane domain that allows its localization at the membranes of late endosomes and lysosomes; in the lysosomal membrane, it interacts with two subunits of the v-ATPase proton pump complex to exert a negative regulation on the amino-acid-dependent mTORC1 activation. In the same paper, the authors demonstrated that loss of SPAR promotes post-injury skeletal-muscle regeneration by increasing mTORC1 activation.

It is noteworthy that all the null mice (Mtln-KO, MLN-KO, SPAR-KO, DWORF-KO) analyzed so far did not show any overt phenotype. The only exception is represented by the Myomixer micropeptide (84 amino acids long), which is generated by a previously annotated lncRNA (Gm7325); germline Myomixer null mice are characterized by perinatal lethality caused by the absence of multinucleated myofibers. This lncRNA has been identified in a loss-of-function screen aimed at the identification of essential genes involved in myoblasts fusion (Bi et al., 2017). Myomixer, also known as minion and myomerger, is essential for muscle formation during embryogenesis, satellite cells fusion, and muscle regeneration (Bi et al., 2017, 2018; Quinn et al., 2017; Zhang et al., 2017); it is embedded in the plasma membrane, and it was suggested to interact with the fusogenic protein Myomaker, thus promoting myoblasts fusion. A more recent paper, in contrast with the previous one, indicate that Myomaker does not need Myomixer to promote hemifusion and that the two proteins are involved in different steps of the fusion pathway (Leikina et al., 2018). Even if the mechanism of action remains to be elucidated, Myomixer role in promoting myoblasts fusion seems to be evolutionary conserved in vertebrates: the Zebrafish ortholog was able to induce heterologous cell fusion when overexpressed with the murine Myomaker (Bi et al., 2017).

## CircRNAs in Skeletal and Cardiac Myogenesis

CircRNAs are originated from a non-canonical splicing reaction, called back splicing, in which a 3′ donor site is unconventionally fused to an upstream 5′ acceptor site. This mechanism generates highly stable and covalently closed molecules lacking a 5′ cap and a poly-A tail and thus inaccessible to the action of cellular exonucleases. Even though the first evidence of their existence goes back to the 1970s, circRNAs have been widely studied only after the advent of next generation sequencing technologies, being recognized as a common element of all metazoan transcriptomes (Wilusz, 2018). *De novo* discovery of circRNAs is based on the presence of exon-junction spanning reads in RNA-seq datasets; the effective existence of a newly identified circRNA then requires PCR validation with divergent primers and RNase R digestion assay to prove exonuclease resistance. This experimental pipeline has allowed the identification and annotation of hundreds of circRNAs, which are expressed and modulated during cultured myoblasts differentiation (Chen et al., 2018) or in developing and aging skeletal muscle of various mammalian species (Li et al., 2017; Wei et al., 2017) (Table 4). The high-throughput discovery of this large amount of circular RNAs has boosted the characterization of their biological significance and the molecular mechanisms of post-transcriptional regulation in which they are involved. A large part of circRNAs show an enrichment for miRNA binding sites in their nucleotide sequence, suggesting a role in competing endogenous RNA networks, which is also corroborated by their high stability. The research of differentially expressed circRNAs between C_2_C_12_ myoblasts and myotubes has identified 581 candidates; according to *in silico* prediction, the top 30 upregulated circRNAs could be part of ceRNA networks involving 91 miRNAs and core myogenic factors like myogenin, Myocyte enhancer factor 2a, myosin heavy chain (Myh)-1, Myh7, and Myh7b (Chen et al., 2018). Similarly, many differentially modulated circRNAs have been identified during several developmental stages of the bovine *longissimus dorsi* skeletal muscle (Wei et al., 2017). Among them, circLMO7, the most downregulated circRNA between adult and embryonic muscles, was shown to positively regulate myoblast proliferation while reducing myoblast apoptosis and differentiation. The biological function of circLMO7 was correlated with its ability to act as a competing endogenous RNA for miR-378a-3p. Starting from the same dataset, the authors demonstrated that other two candidates impact the development of bovine skeletal muscle through a miRNA-binding activity. Specifically, CircFUT10 was observed to promote myoblasts survival and differentiation and to reduce cell proliferation rate by directly binding to and inhibiting miR-133a; in this way, the circRNA upregulates the serum response factor downstream targets, resulting in a positive impact on myogenesis (Li et al., 2018a). On the other side, CircFGFR4 promotes bovine primary myoblasts differentiation by sequestering miR-107 and thus derepressing its known target Wnt3a, a key upstream factor of the Wnt/β-catenin signaling pathway (Li et al., 2018b). Recently, circSNX29 was identified as another player in the regulation of bovine skeletal muscle development (Peng et al., 2019). This molecule facilitates myoblast differentiation by sponging miR-744 to attenuate its inhibitory effect on the Wnt5a/Ca^2+^ signaling pathway.

**Table 4:** Circular RNAs (circRNAs) in skeletal and cardiac myogenesis.

**circRNA**	**Species**	**Function/mechanism**	***In vivo* model phenotype**	**Expression**	**References**
circLMO7	Bovine	ceRNA for miR-378a-3p to positively regulate myoblast proliferation	Not available	Highly expressed in muscle	Wei et al., 2017
circFUT10	Bovine	ceRNA for miR-133a to promote myoblast survival and differentiation by upregulating the serum response factor downstream targets	Not available	Highly expressed in muscle	Li et al., 2018a
circFGFR4	Bovine	ceRNA for miR-107 to promote myoblast differentiation by de-repressing Wnt3a	Not available	Highly expressed in muscle	Li et al., 2018b
circSNX29	Bovine	ceRNA for miR-744 to promote myoblast differentiation by derepressing Wnt5a/Ca^2+^ pathway	Not available	Highly expressed in muscle	Peng et al., 2019
circSVIL	Chicken	ceRNA for miR-203 to promotes the proliferation and differentiation of myoblasts	Not available	Highly expressed in leg muscle	Ouyang et al., 2018
circ-ZNF609	Mouse, human	Regulates myoblast proliferation and contains an open reading frame that can be translated; ceRNA for miR-194-5p to inhibit myogenic differentiation	Not available	Expressed in a wide range of tissues	Legnini et al., 2017; Rossi et al., 2019; Wang Y. et al., 2019
circMbl	*Drosophila*, human	Encodes for a protein to regulate the splicing of its own host gene	Motorial defects and peculiar wing position	Expressed in muscle and brain tissues	Pamudurti et al., 2017, 2018
circ-Ttc3	Mouse, rat	ceRNA for miR-15b-5p to increase the expression of Arl2	Deterioration of cardiac dysfunction after MI	Highly expressed in heart	Tan et al., 2017; Cai B. et al., 2019
circSlc8a1	Mouse, rat, human	ceRNA for miR-133a to regulate its targets	Attenuates cardiac hypertrophy from pressure overload	Highly expressed in heart	Werfel et al., 2016; Lim et al., 2019
circMFACR	Mouse	ceRNA for miR-652-3p to downregulate MTP18 at the translational level and favors mitochondrial fission and apoptosis	Not available	Highly expressed in heart	Wang et al., 2017
circNfix	Mouse, rat, human	Induces Ybx1 degradation by ubiquitination; ceRNA for miR-214 to promote Gsk3β expression and to repress β-catenin activity	Increases in cardiomyocyte proliferation	Highly expressed in cardiomyocytes	Huang et al., 2019

Moreover, the analysis of circRNA expression during chicken skeletal muscle development identified circSVIL. circSVIL promotes the proliferation and differentiation of myoblasts by binding and antagonizing the function of miR-203, a post-transcriptional repressor of c-JUN and MEF2C (Ouyang et al., 2018). Furthermore, a massive high-throughput screening of both proliferating and differentiated human primary myoblasts and mouse C_2_C_12_ cells revealed an abundant population of cytoplasmic circRNAs, which showed a global accumulation during differentiation (Legnini et al., 2017). Interestingly, a large part of them was found deregulated in human myoblasts derived from Duchenne muscular dystrophy patients. A subset of highly conserved, strongly modulated circRNAs was selected for a wide phenotypic screening, from which circ-ZNF609 emerged as a regulator of myoblast proliferation, while Circ-QKI and circ-BNC2 showed a positive and negative effect on myogenesis, respectively. Of them, Circ-ZNF609 contains an ORF that can be translated in a cap-independent manner due to the IRES activity of the untranslated region (Legnini et al., 2017). More recently, it has been shown that CircZNF609 is upregulated in rhabdomyosarcoma biopsies and that its knockdown induces a specific arrest of G1/S transition rhabdomyosarcoma-derived cells. The cell cycle block is the result of a strong decrease in phosphorylated Akt protein level and an alteration of the p-Rb/Rb ratio (Rossi et al., 2019). The role of this molecule in tumor progression has been validated also in breast cancer, where high levels of Circ-ZNF609 are associated with poor prognosis (Wang S. et al., 2018). Moreover, the mouse homolog of CircZNF609, named circZfp609, has been shown to inhibit myogenic differentiation by sponging miR-194-5p (Wang Y. et al., 2019).

Circ-ZNF609 is only one of the examples of a protein-coding circRNA in eukaryotes (Chekulaeva and Rajewsky, 2019). The Muscleblind locus encodes for a splicing factor with a critical function in muscle development; its sequestration from aberrant CUG repeats is causative of myotonic dystrophy (Miller et al., 2000). In *Drosophila*, this locus produces also circMbl, an abundant protein-coding circRNA that regulates splicing of its own host gene (Pamudurti et al., 2017). Interestingly, circMbl knockdown in the whole organism has a specific effect on muscle development and function, as confirmed from gene expression analysis, motorial defects, and a peculiar wing position (Pamudurti et al., 2018). In their latest work, Chen et al. identified a subset of 224 circRNAs modulated during C_2_C_12_ differentiation with *bona fide* coding potential according to the number of open reading frames and N6-methyladenosine motifs, which are known to work as promoting factors for cap-independent translation (Chen et al., 2018). It was recently suggested that circular RNAs expression can be affected by splicing alterations related to myotonic dystrophy type 1 (DM1), a multisystemic disorder in which expanded CTG repeats in the DMPK gene leads to splicing abnormalities. CircRNAs expressed in DM1 skeletal muscles biopsies were identified by analyzing RNA-sequencing datasets, demonstrating the upregulation of CDYL, HIPK3, RTN4_03, and ZNF609 compared to healthy controls (Voellenkle et al., 2019). The circular fraction values were positively correlated with splicing biomarkers of disease severity, reaching higher values in more severely affected patients. Measurement of circular-to-linear ratios for these candidates resulted to be a good prediction method to discriminate DM1 patients from controls, suggesting a possible future use as biomarkers.

The miRNA-binding paradigmatic mechanism has been validated also in cardiac muscle. In a recent study, Tan et al. performed a deep RNA-sequencing from human and mouse hearts and across a differentiation time-course of human embryonic stem-cell-derived cardiomyocytes. They identified a total of 15,318 and 3,017 cardiac circRNAs within human and mouse, respectively. Among them, circ-Ttc3 resulted in one of the top highest expressed circRNAs in mouse heart (Tan et al., 2017). Cai L. et al. (2019) found that circTtc3 was markedly upregulated in the ischemic myocardium, whereas there was no significant change in linear Ttc3 RNA. Overexpression of circ-Ttc3 in cardiomyocytes counteracted hypoxia-induced ATP depletion and apoptotic death. Mechanistically, this circRNA sponges and inhibits miR-15b-5p leading to the increased expression of its target Arl2. Consistently, Arl2 knockdown partially abolished the beneficial effects of circ-Ttc3 overexpression on ATP production and apoptosis of cardiomyocytes. Thus, the Circ-Ttc3-miR-15b-Arl2 regulatory cascade may have a cardioprotective role in myocardial infarction (Cai L. et al., 2019). The same screening allowed the identification of circSlc8a1, which is the most abundant single-exon cardiac expressed. A new study demonstrated that circSlc8a1 inhibition attenuated cardiac hypertrophy, while its forced overexpression resulted in heart failure. As reported for the majority of the circRNAs identified so far, also this circular molecule act in the cytoplasm by sponging a miRNA, in particular the cardiac-enriched mir-133a. Considering that the overexpression of miR-133a attenuates cardiac hypertrophy both *in vitro* and *in vivo*, inhibiting circSlc8a1 may result as a novel therapeutic target for cardiac hypertrophy (Lim et al., 2019). An increasing number of studies have indicated that mitochondrial fission dysfunction occurs in many cardiac diseases, such as MI and heart failure. Recently, it has been suggested that circRNAs could play a role also in this context. In this regard, CircMFACR favors mitochondrial fission and apoptosis in the heart by sequestering miR-652-3p, which in turn downregulates MTP18 expression at the translational level. MTP18 is a nuclear-encoded mitochondrial membrane protein whose deficiency reduces mitochondrial fission and suppresses cardiomyocyte apoptosis and MI. Thus, the silencing of MFACR expression leads to a reduction in mitochondrial fragmentation and cell apoptosis. This study revealed that circRNAs can play an active role in the regulation of mitochondrial dynamics, cardiomyocyte apoptosis, and myocardial infarction (Wang et al., 2017). CircRNAs were found to regulate cardiac regeneration. One of the most recent example is circNfix, a superenhancer-regulated circular RNA whose depletion produces cardiomyocyte proliferation and angiogenesis. RNA pulldown and luciferase reporter assays revealed that circNfix acts both as a miR-214 sponge and as a destabilizing partner of Ybx1 *via* ubiquitin-dependent degradation (Huang et al., 2019).

## piRNAs and tRFs in Skeletal and Cardiac Myogenesis

The regulatory potential of the non-protein-coding transcriptome is not limited to the better characterized lncRNAs but includes also less known and more recently discovered RNA species. The piRNAs are a class of small RNAs widely conserved in all the metazoans. Following transcription and processing, the mature piRNA form associates with different members of the PIWI proteins clade, thus creating silencing molecular complexes that defend the germline genome from transposon expression by acting at both transcriptional and post-transcriptional level (Ozata et al., 2019). The common perception of the PIWI–piRNA pathway as a germline-specific feature is nowadays questioned by the evidence of expression of this molecular machinery in a variety of somatic tissues (Lee et al., 2011; Perera et al., 2019). While the functional role of somatic piRNAs is still debated in other tissues, recent works suggest that these small RNAs could regulate heart regeneration due to their effect on the Akt pathway, a fundamental network in heart physiopathology. Microarray profiling of different populations of cardiac progenitors identified a consistent set of piRNAs, which are differentially modulated between cardiospheres, cardiosphere-derived cells, and cardiac fibroblasts, thus creating a specific molecular signature of these cell populations (Vella et al., 2016). In particular, piRNAs which are specifically upregulated in cardiosphere-derived cells act as negative regulators of L1 retrotransposon transcripts, whose inhibition has been shown to decrease upon post-ischemic myocardial damage by the activation of the Akt signaling (Lucchinetti et al., 2006).

**Figure 1:**
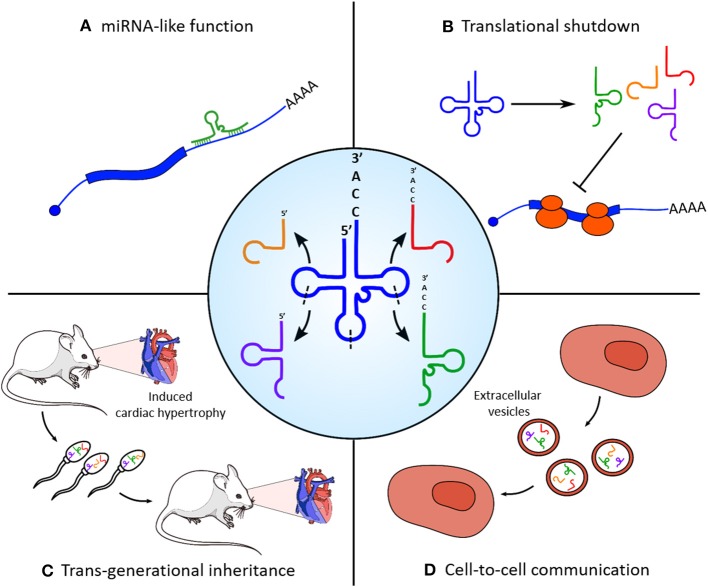
Functional roles of tRNA-derived fragments (tRFs) in skeletal and cardiac muscle homeostasis. Central panel: tRNAs are processed into different categories of tRFs by enzymatic cleavage on specific sites (indicated with dashed lines). **(A)** tRNA fragments can target the 3′ untranslated region (UTR) of protein-coding transcripts (e.g., Kassiri et al., 2009) and regulate their stability and translation efficiency with a microRNA (miRNA)-like mechanism. **(B)** Massive cleavage of specific tRNA species in stress conditions induces a rapid translational blockade. **(C)** Accumulation of tRFs in the sperm is responsible for the non-genetic transmission of myocardial hypertrophy phenotypic traits in the offspring. **(D)** tRFs produced from myoblasts are selectively loaded in extracellular vesicles and could act as mediators of cell-to-cell communication.

tRNAs represent another class of small RNAs which are emerging as regulators of heart and skeletal muscle physiology (Figure 1). They are well-known for their canonical function as adaptor molecules during protein synthesis; however, it has been recently discovered that their controlled endonucleolitic cleavage could represent a source of small regulatory non-coding RNAs. These small molecules were first identified during miRNA library cloning experiments (Fu et al., 2005) and initially classified as random tRNA degradation misproducts; however, the conserved length and the recurrent position of cleavage sites suggested the existence of a dedicated enzymatic activity responsible for their biogenesis. tRNA fragments can be grouped in three categories (Liapi et al., 2019), based on their derivation from the 5′ or 3′ end of parental tRNA (5′/3′ tRFs), from the 3′ portion of the immature tRNA precursor (tRF1s), or from a cleavage event within the anticodon loop which splits the parental molecules in two 30–35 nucleotide long (5′ and 3′) halves. tRNA halves biogenesis is an evolutionary widespread molecular event of particular interest, since they tend to be produced as a response to several cellular stresses by the activity of the angiogenin (ANG) endonuclease. The first report of such mechanism in mammals has been made in tissues of various origin, including *ex vivo* starved mouse hearts, which showed a specific accumulation of the 5′ portion of Val-AAC tRNA (Fu et al., 2009). The accumulation of a small subset of tRNA halves compared to the complexity of parental tRNA pools corroborates the hypothesis of a controlled processing machinery and of a functional role for tRNA fragments, other than a simple mechanism of stress-induced translational shutdown. Moreover, the expression of specific tRNA fragments was found to be strongly upregulated in a rat model of induced myocardial hypertrophy (Shen et al., 2018); interestingly, transfection of a synthetic version of the two most upregulated tRFs in cultured cardiomyocites increases both hypertrophy marker expression and cell surface area. In particular, Kassiri et al. (2009) show that tRFs1, which derives from tRNA-Gly-GCC cleavage, targets the 3′ untranslated region of Timp3, a well-known cardiac hypertrophy regulatory factor in a miRNA-like manner. Remarkably, mice with induced myocardial hypertrophy had high levels of the identified tRFs also in sperm; a similar pattern of expression of these fragments was found in offspring hearts, which showed an intermediate phenotype with extended heart muscle fiber breakage and fibrosis. Taken together, these findings not only indicate a role for tRFs in the physiopathology of heart but also their involvement on the transgenerational inheritance of a non-genetic stress condition, as already reported for sperm-contained miRNAs (Rodgers et al., 2015).

Recent works have shown that small RNAs are actively loaded into extracellular vesicles (EVs) and released in almost all kind of biofluids to act as mediators of cell-to-cell communication (Tkach and Théry, 2016). Even if this phenomenon has been widely studied for miRNAs, there are also evidences of exosome-mediated secretion of other small RNA species, such as piRNAs and tRNA fragments, from different tissue sources (Chiou et al., 2018; Tosar et al., 2018). Of note, the small RNA transcriptome from both human rhabdomyosarcoma RD4 and murine C_2_C_12_ myoblasts and of EVs produced by these cells has been recently analyzed (Sork et al., 2018). It emerged that small RNAs are underrepresented in EVs compared to their parental cells, suggesting a loading mechanism that is not explicable with simple diffusion. Moreover, C_2_C_12_-produced EVs present a strong enrichment of piRNAs and tRNA fragments compared to intracellular content, including the tRNA-Gly-GCC fragment cited above. Taken together, these evidence suggest that these small RNAs can be selectively included in secretory vesicles, with a potential role in long distance cell-to-cell communication events.

High-throughput small RNA sequencing from four types of wild-type and dystrophic (*mdx*) muscles and from sera allowed to discover that many piRNAs and tRFs are expressed in skeletal muscle tissue and differentially released in the circulation in dystrophic conditions. Furthermore, the high level of piR_000620 and piR_000935 detected in *mdx* serum was restored to nearly wild-type levels in response to DMD exon skipping with injected modified oligonucleotides (Coenen-Stass et al., 2018). The differential release of piRNAs has been also observed in serum vesicles isolated from heart failure patients compared to healthy donors, suggesting a possible use of these small RNAs as clinically relevant biomarkers (Yang et al., 2018).

## Author Contributions

JM wrote the manuscript and selected the literature. DM and FD reviewed the manuscript and prepared tables and figures. MB proposed the topic, wrote the manuscript, and reviewed the text.

### Conflict of Interest

The authors declare that the research was conducted in the absence of any commercial or financial relationships that could be construed as a potential conflict of interest.
